# Nonmechanical parfocal and autofocus features based on wave propagation distribution in lensfree holographic microscopy

**DOI:** 10.1038/s41598-021-81098-7

**Published:** 2021-02-05

**Authors:** Agus Budi Dharmawan, Shinta Mariana, Gregor Scholz, Philipp Hörmann, Torben Schulze, Kuwat Triyana, Mayra Garcés-Schröder, Ingo Rustenbeck, Karsten Hiller, Hutomo Suryo Wasisto, Andreas Waag

**Affiliations:** 1 Institute of Semiconductor Technology (IHT), Technische Universität Braunschweig, Hans-Sommer-Straße 66, 38106 Braunschweig, Germany; 2 Laboratory for Emerging Nanometrology (LENA), Technische Universität Braunschweig, Langer Kamp 6, 38106 Braunschweig, Germany; 3 Faculty of Information Technology, Universitas Tarumanagara, Jl. Letjen S. Parman No. 1, Jakarta, 11440 Indonesia; 4 Institute for Biochemistry, Biotechnology and Bioinformatics, Braunschweig Integrated Centre of Systems Biology (BRICS), Technische Universität Braunschweig, Rebenring 56, 38106 Braunschweig, Germany; 5 Institute of Pharmacology, Toxicology and Clinical Pharmacy (IPT), Technische Universität Braunschweig, Mendelssohnstraße 1, 38106 Braunschweig, Germany; 6 Department of Physics, Faculty of Mathematics and Natural Sciences, Universitas Gadjah Mada, Sekip Utara, PO Box BLS 21, Yogyakarta, 55281 Indonesia

**Keywords:** Optical imaging, Interference microscopy, Imaging and sensing, Computer science

## Abstract

Performing long-term cell observations is a non-trivial task for conventional optical microscopy, since it is usually not compatible with environments of an incubator and its temperature and humidity requirements. Lensless holographic microscopy, being entirely based on semiconductor chips without lenses and without any moving parts, has proven to be a very interesting alternative to conventional microscopy. Here, we report on the integration of a computational parfocal feature, which operates based on wave propagation distribution analysis, to perform a fast autofocusing process. This unique non-mechanical focusing approach was implemented to keep the imaged object staying in-focus during continuous long-term and real-time recordings. A light-emitting diode (LED) combined with pinhole setup was used to realize a point light source, leading to a resolution down to 2.76 μm. Our approach delivers not only in-focus sharp images of dynamic cells, but also three-dimensional (3D) information on their (*x, y, z*)-positions. System reliability tests were conducted inside a sealed incubator to monitor cultures of three different biological living cells (i.e., MIN6, neuroblastoma (SH-SY5Y), and *Prorocentrum minimum*). Altogether, this autofocusing framework enables new opportunities for highly integrated microscopic imaging and dynamic tracking of moving objects in harsh environments with large sample areas.

## Introduction

Nowadays, continuous monitoring of morphological change and motion in living cell culture is typically performed by direct imaging using a conventional optical microscope installed into a sealed incubator^1–6^. However, the available setups are limited by the requirements of lenses with a complex configuration. This leads to a fixed focal length, a small field-of-view, a short working distance, bulky, expensive instruments, and difficult integration into incubators. Compact conventional microscopes often do not reach the optical quality of large optical systems in terms of focal lengths, magnifications, and optical aberrations, limiting their functionality^7,8^. In addition, complex optical lens systems often lead to inhomogeneous resolution across the overall image, which is very sharp at the image center, but decreasing towards the edges. This may cause a problem to determine the shape and position of a moving object of interest. Recently, several microscope manufacturers (e.g., Tokai Hit Co., Ltd.) and research groups have attempted to build optical microscopes with an integrated mini incubator that is specifically designed to enable live-cell monitoring and analysis^9,10^. Despite their good functionality, those incubator-combined microscopes still face several limitations (e.g., less configuration flexibility, small incubator size, and high cost).

Lensless holographic microscopy emerges as a promising alternative to its conventional lens-based optical counterparts in life sciences, reaching a similar resolution while still being mobile, cost-effective, robust, and reliable for biomedical applications. It can be easily customized to be used with standard sample slides, petri dishes or specific microfluidics, making this lensfree imaging system advantageous over conventional table-top optical microscopes^11,12^, especially in terms of simultaneous parallel investigation and usage in harsh environment settings (i.e., incubators with high temperature and humidity). It creates a compact setup with a short sample-to-sensor distance and an equally sharp reconstructed image at all positions across a large field-of-view^13^. In contrast to lens-based systems, a holographic interference pattern is captured in the lensfree setup instead of an already focused image of the object. To solve the image reconstruction problem, a digital holographic image reconstruction based on numerical approaches (e.g., angular spectrum^14^ and Fresnel diffraction methods^15,16^) can be implemented to back-propagate the recorded interference pattern into the correct *z*-position. The raw diffraction image of the illuminated sample is transformed by computational reconstruction into a wave representation of the sample.

Although different designs and approaches of lensless digital holographic microscopes have been reported to be used in a wide range of biomedical and environmental monitoring applications^14,17^, they have not employed statistical approaches (e.g., skewness, chi-square, power-divergence, Jarque–Bera, kurtosis, normality test, and Gini index) for analyzing the wave propagation distributions. Moreover, the benefits of the parfocal function in lensless microscopy were also not described. From the other report, a lensfree imaging system was integrated with microfluidics for point-of-care testing^12^. The captured images were however not reconstructed with either manual or automatic process. Therefore, besides the unsatisfactory interference pattern, the resulting image has poor quality. In that setup, a charge-coupled device (CCD) sensor was utilized instead of a complementary metal oxide semiconductor (CMOS) image sensor. It is known that even though CCD technology has been dominant in the market for visible photon detection and image acquisition over the last two decades, it has several drawbacks (e.g., slow serial access to image for large-size arrays, high power dissipation because of intrinsic capacitive nature of gates, and high power consumption)^18^. Thus, CMOS detectors are preferable to be used in microscopy application due to their intrinsic advantages (i.e., low power consumption, high readout rate, low noise, and high integration capability down to the pixel level)^18,19^.

Previous studies mostly demonstrated that cell detection in holographic microscopy could only be performed on clear shadow images, where the small size and high coherence degree of the light source possessed a key role to reduce the geometric unsharpness^20–23^. Here, lasers have been commonly used in holographic system because of their high temporal and spatial coherence^24,25^. However, a too high coherence can also result in enormous speckle noise lowering the produced image quality^26^. Thus, a light-emitting diode (LED), has been selected to tackle the speckle problem and similar interference issues^26,27^. The emission of an LED is incoherent in phase, but coherent in space, depending on its geometrical dimension. Therefore, attaching a pinhole structure on the LED is suitable method to yield an ideal light source for lensless holographic imaging.

In respect to the base material, gallium nitride (GaN)-based LED has been widely accepted and employed as a promising solid-state light source because of its high efficiency, robust structure, high brightness, fast modulation, and long lifetime. These are achieved not only for large-area high power LEDs used in solid state lighting, but also for LEDs with dimensions of below 10 µm (i.e., microLEDs) as structured micro-illumination sources^28–32^. To support the development of the compact microscope, micro- and nanoscale GaN-based LEDs can be fabricated using either bottom-up or top-down approach, as well as processed to be a highly integrated pinhole microLED array, which is beneficial for increasing spatial coherence and hence the imaging capability^22,33–36^.

Despite its simple mechanical configuration, the extraction of a real image from the diffraction pattern faces several challenges. This contains the shadowing of structures, the irregular background intensity, and the appearance of out-of-focus interference patterns that hinder the distinguishability between sample structures and background noise^37^. Moreover, the image reconstruction process requires the *z*-distance between the sensor and the sample as an input parameter, which is still estimated either manually or by complex and time-consuming algorithms^38–41^. To address these issues, the autofocus feature^42–45^ and parfocal technique described in this work can be employed simultaneously. The former is dedicated to automatically and instantly find the sharpest image possible at all times, while the latter is assigned to keep the object of interest staying in-focus when the focal length changes due to object movement in *z*-direction or alteration in magnification level^46–48^. The parfocal system is an important building block in many imaging instruments and can be commonly found in bright-field microscopes. Nonetheless, for the conventional parfocal technique, a complicated setup combining special objectives and several magnifying lenses are required to obtain the in-focus effect. This leads to several drawbacks, i.e., higher cost for setup realization, difficult incorporation into specific applications, and time-consuming process for long-term and continuous experiments^49^. To perform an automatic focus tracking, Ferraro et al. have developed a digital holographic microscope consisting of a 532 nm laser light source and an objective with specific focal length and numerical aperture^23^. Despite its success in the phase-shifting measurement of holographic interference fringes, the microscope has not been tested for tracking biological cells. Moreover, because of its complex and bulky setup, the system was not suitable for being used inside a conventional incubator to perform a real-time cell monitoring.

Lately, several statistical and computational methods have been proposed to evaluate the focus quality in lensless holographic microscopy reconstructions. Among other techniques (e.g., non-linear correlation, power spectrum, variance, summed gradient, Laplacian, and high-frequency discrete cosine transform^50–53^), a method based on the Gini index has become one of the most popular methods to deliver the best in-focus position^54–56^. Nevertheless, this algorithm requires pre-processing steps in form of background subtraction and edge detection, increasing the required processing time^41^. Besides, with the rise of interest to create smarter imaging systems, self-learning algorithms like deep learning and machine learning were also investigated lately regarding their autofocus usage^41,57^. However, again, a complex computational architecture, which requires a lengthy and arduous training process on image recognition, should firstly be performed before the autofocus can be used on the object of interest, resulting in a longer processing duration.

Hann-filter algorithm was also reported to be used to find the best focus values of the images through an interval search approach^25^. It could perform three-dimensional (3D) tracking of moving cells. However, again, the presented holographic imaging system still employed lens and laser light source. In another lens-based microscope, a single-shot mechanical autofocusing method was demonstrated for fluorescence imaging, in which a motorized stage was employed to move the sample position and keep the image always in focus^45^. The critical sampling of the complex field was used to improve the processing speed by adjusting the holographic image sampling size. The main drawbacks of this approach are its slow response and low accuracy during the autofocusing operation, which originated from the limited speed and high slip in the used motor.

In this work, we developed a novel parfocal autofocusing method for real-time imaging in a pinhole LED-based lensless holographic microscope. Instead of using complex and expensive laser light source that has several issues (e.g., high speckle noise and adverse effect on both biological cells and CMOS sensor), low-power LED was utilized as a light source to illuminate our sample, in which a pinhole plate was added to improve its spatial coherence and to produce optimum interference pattern of the holographic image. The function of a parfocal lens was substituted with a computational analysis of the wave propagation distribution from the sampled dataset, which was performed by calculating the similarity of the intensity distribution to the Gaussian profile and analyzing the asymmetrical statistical distribution, resulting in a continuous autofocus condition. Due to the computational holographic reconstruction, any mechanical adjustment of microscope parts could be completely omitted, i.e., to adjust the lens or sample-to-objective-lens distance. We explored the usage of various light sources and statistical approaches (i.e., skewness, chi-square, power-divergence, Jarque–Bera, kurtosis, normality test, and Gini index) to find the optimum image quality and obtain a faster processing speed of the information from the raw hologram images. Besides, to investigate its reliability and performance, the system was directly tested inside a sealed incubator for a live monitoring of different cell cultures. Our parforcal autofocusing holographic microscope was not only capable of performing long-term observation (for more than seven days), but also providing a 3D position information of the moving cells (in *x*-, *y*-, and *z*-directions) while maintaining in-focus images automatically.

## Results and discussion

### Light source optimization based on spatial coherence

**Figure 1:**
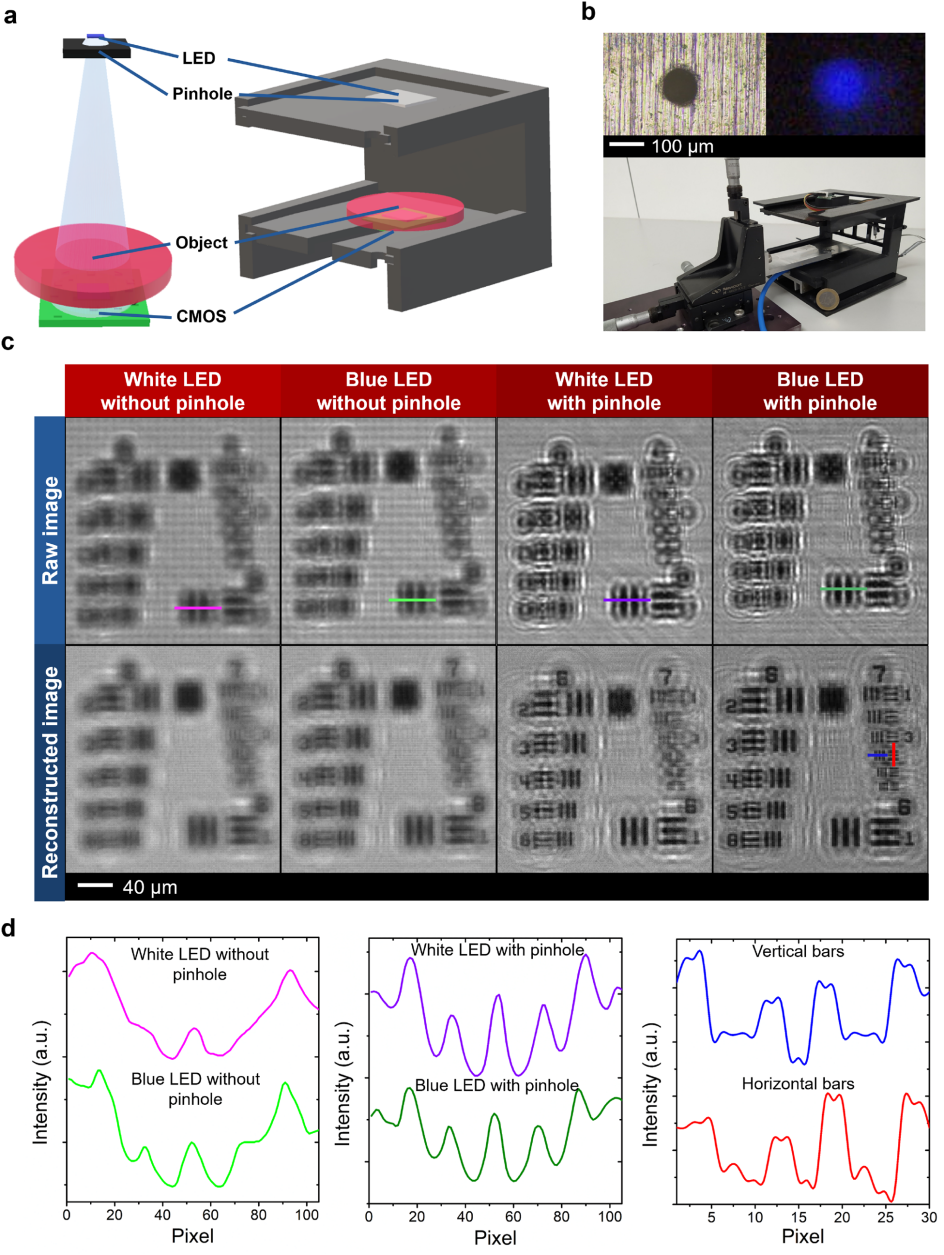
Portable LED-based lensless microscope and its limit of resolution in pinhole and non-pinhole configurations. (**a**) Schematic of digital in-line holographic microscope consisting of a light-emitting diode (LED), a pinhole, a specimen, and a complementary metal oxide semiconductor (CMOS) image sensor. 3D visualization was obtained using Paint 3D (Microsoft Corporation). (**b**) Point light source produced by a blue LED emitting at a wavelength of 467 nm and a covering pinhole of 100 μm used in a lensless microscope coupled with an *x–y–z* micrometer stage. (**c**) Raw and reconstructed intensity images using white and blue LEDs with and without pinhole setup. The resolved resolution limit is measured by the smallest recognizable distance in the USAF test pattern. Python 2.7.14 (https://www.python.org) and Opencv 3.4.1 (https://opencv.org) were used to generate the images. (**d**) Line scan over the first element of group 6 in the USAF chart produced by different LEDs with and without pinhole setup. The spatial and temporal coherences affected the achieved system resolution, where the resolved resolution limit is shown from the element 4 of group 7 after image reconstruction. OriginPro 2019b was employed for plotting the figures (https://www.originlab.com).

The main goals of this study are to obtain the sharpest holographic image and to continuously keep it in-focus while the object position changes, especially in *z*-direction. Instead of employing a laser as the light source, an LED was integrated into the lensfree microscope (Fig. 1a,b) mainly because of its partial coherence characteristic, stable intensity, and harmlessness to living samples or CMOS image sensors^28,58^. However, the spatial coherence of the LED-illumination has to be adjusted to reduce the geometric unsharpness. The coherence of the light source can generally be divided into temporal and spatial coherences. The former is related to phase coherent emission in combination with the spectral bandwidth of the light source, while the latter describes the phase uniformity of a wavefront in space by analyzing its space correlation and ability to produce stable interference patterns^59^. As the spatial coherence can be also influenced by LED size and propagation distance, integrating a microscale pinhole (e.g., with a hole diameter of < 300 µm) onto an LED as a spatial filtering component can increase the spatial coherence and consequently the imaging capability^26^.

We tested both polychromatic and nearly monochromatic LEDs (i.e., white and blue LEDs) having conditions of with and without pinhole to find the optimum light source for the later experimental setup by comparing the clarity and sharpness of the reconstructed images obtained from the United States Air Force (USAF) 1951 resolution test target (Fig. 1c,d). On this test chart, nine patterned groups with different sizes are available, in which each group comprises six elements of vertical and horizontal black bars with precisely defined width and spacing. This target was used to determine the best in-focus position and the resolution of the imaging system, which was indicated by the clearly distinguished space between the two smallest possible bars. The raw images captured by the CMOS sensor were reconstructed for various *z*-positions using an angular spectrum algorithm (see “Methods” section), while the wave propagation distribution analysis was used to find the best focal position and the sharpest image within several *z*-planes.

A high spatial coherence of the object illumination is required to produce a distinct diffraction pattern with a high resolution. Otherwise, only a blurry random pattern will be formed. Using a white LED, the interference pattern formed on the CMOS active area is produced by a range of wavelengths^59^. Therefore, the illumination led to a mixture of several fringes from different wavelengths, which can increase the pattern unsharpness (Fig. 1c,d). In lensless imaging, samples with a high object density and narrow object spacing can result in a low quality of the reconstructed image due to the phenomenon of diffraction limit^60^. As the light wave enters the aperture between the edges of objects with very small distance, the diffracted waves will interfere. This interference like the one appearing from the first element of group 6 in the USAF chart can be either constructive or destructive (Fig. 1c,d).

The reduced spatial coherence length of the light sources without pinhole leads to unclear interference patterns (Fig. 1c columns 1 and 2), indicating that the center of the Gaussian spot has been increased leading to a lower system resolution and a reduced noise pattern. The experimental results demonstrated that pinhole-covered LEDs possessed a higher spatial coherence compared to the bare LEDs. The pinhole could significantly decrease the aberration of the diffraction pattern from the USAF target (Fig. 1d). However, it also simultaneously reduced the light intensity that was used to expose the object. To mitigate this limitation, a shorter light-source-to-sample distance and a post-image processing could be opted and performed, respectively, resulting in better image intensity. Again, it is clear from the comparison of the interference patterns produced by non-coherent (without pinhole) and partially coherent (with pinhole) light sources that the clearest images with the highest resolution were obtained when the pinhole-covered blue LED was used in the setup. This could be proven by comparing the horizontal and vertical profiles in the fourth element of group 7 in the USAF (indicated in blue and red lines, respectively, in Fig. 1c,d). With this light source configuration, the lensless microscope was able to observe the sample with a size of down to 2.76 μm, which is considered as our microscope resolution (Fig. S1 in Supplementary Information). Moreover, the fifth element of group 7 in the USAF with a size of 2.46 μm was still recognizable, but not very clear (Figs. 1c and S1). These results indicate that using a blue LED with a pinhole can reach the image resolution that is very close to the limit of the CMOS pixel size (2.2 μm), which however, is only of relevance when the object is positioned directly on top of the CMOS sensor. Thus, from this point onward, we used a blue LED with pinhole setup as the light source for further measurements with the lensfree microscope.

A 100 µm pinhole was used to limit the light emission from the 150 µm × 200 µm LED source. The optical power densities of the blue LED before and after being integrated with the pinhole are 298 and 0.257 µW/cm^2^, respectively. To analyse the temporal coherence of our setup, we compared the measured spectra of the blue LED with and without pinhole (see Fig. S6a in Supplementary Information). It was found that both spectra possess similar shape with a peak wavelength of 467 nm, which indicates the unchanged temporal coherence. However, in case of the pinhole LED, a lower illumination intensity was yielded.

### Autofocus and parfocal features

**Figure 2:**
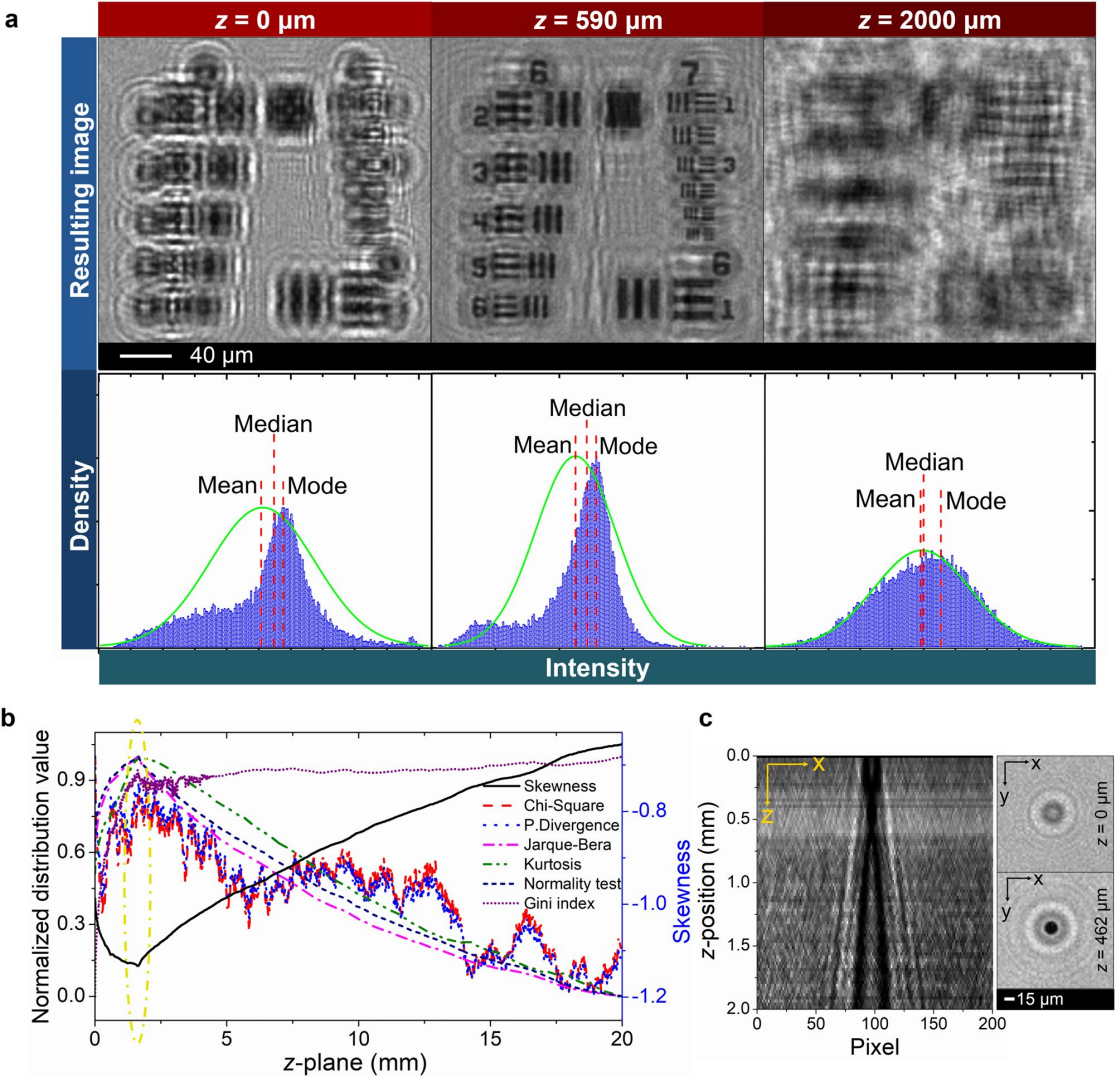
Wave propagation distribution analysis. (**a**) Enlarged view of groups 6 and 7 from the calculated distances of *z* = 0 μm (input image), 590 μm, and 2000 μm, respectively. The first-order histograms (blue) including their parameters (mean, median, and mode) compared to normal distribution curves (green) show that the best in-focus position of the USAF target sample is found at *z* = 590 μm, where the largest difference between the histogram and normal distribution occurs. (**b**) Different distribution analysis criteria of USAF sample along various *z*-planes. The yellowish dashed circle exhibits the best in-focus position at *z* = 1.63 mm above the sensor. (**c**) Micro dot image at *z* = 0 and reconstructed dot image at *z* = 462 µm. The point spread function (PSF) shows the scanning over *z*-position and the limited resolution in the axial direction. USAF and PSF figures were created using Python 2.7.14 (https://www.python.org) and Opencv 3.4.1 (https://opencv.org), while intensity and normalized distribution analysis figures were plotted using OriginPro 2019b (https://www.originlab.com).

After optimizing the light source used in the setup, we then placed a USAF test target directly above the sensor in the microscope to start developing the autofocus and parfocal features. As a result, the interference patterns could be seen clearly, making the USAF pattern unrecognizable in the interference pattern (Fig. 2a; *z* = 0 μm). This phenomenon is due to the fact that the interference pattern was recorded at *z* = 0, which is the plane of the CMOS sensor device. Nonetheless, the actual object position was at an unknown *z*-position, given by the gap between the active layer of the photodiode inside the CMOS sensor and the outer edge of the sample surface, as well as the glass layer thickness of the USAF sample. Thus, the wave propagation distribution analysis was employed to find the right *z*-plane for the reconstruction of the images. The highest resolution was perfectly solved by combining the angular spectrum method and wave propagation distribution analysis to back-propagate and find the correct *z*-position of the sample at the position, where the “clearest” reconstructed image occurred (Fig. 2a; *z* = 590 μm). When the *z*-plane of the reconstruction has been further shifted up to *z* = 2000 μm, the yielded USAF pattern image blurriness increased again, due to the large discrepancy between the supposed and actual positions. Identifying the sharpest image does not only represent the highest quality of the image, but also provides an estimate of the distance between sensor and object. The *z*-value is a parameter in the angular spectrum algorithm for calculating the back propagation of the image to the object plane. While moving mathematically in the *z*-direction, the wave propagation distribution analysis calculates the distribution of intensity to find the highest non-normal distribution (as compared to the intensity distribution of input images) automatically without requiring any pre-processing step. The first-order histogram parameter assessment was conducted in the distribution analysis. Our automated algorithm demonstrated a significant increase in sensitivity, specificity, and accuracy in perceiving the best in-focus image.

The first-order histogram parameters exhibit various intensity distributions from the raw (*z* = 0) and reconstructed images (Fig. 2a). The blue bars and green lines indicate the intensity histogram and the normal distribution model obtained from its intensity, respectively. An obvious dissimilarity between the normal distribution and histogram can be seen in Fig. 2a, while the skewed histogram difference also appears in different *z*-planes. To prove this, a single 15 μm dot image was reconstructed by conducting a rough *z*-scan ranging from 0 to 2 mm with a step size of 10 μm (Fig. 2c). As a result, the best focal position of *z* = 462 µm was found from a narrow area in the Gaussian curve, obtaining the highest resolution in the *z*-propagation.

The basic concept for the autofocus feature is to analyze the density and distribution of pixel intensity from the histograms, where the sharpness and blurriness in the images can be differentiated (Fig. S2 in Supplementary Information). In general, the wave propagation distribution was measured using the intensity distribution as input data. Here, the result was zero if all the data were identical with the Gaussian curve (Fig. 2a). Meanwhile, it would increase in number as the data became more diverse.

A key challenge is the identification of the “best” image, giving at the same time the “correct” z-position of the object plane. Here, we extracted quality parameters from the back-propagated image for a given (but changing) z-position based on various statistical approaches for analyzing the wave propagation distributions (i.e., skewness, chi-square, power-divergence, Jarque–Bera, kurtosis, normality test, and Gini index). All these statistical approaches were performed on the USAF pattern images to identify the best and most stable procedure.

Skewness is an asymmetrical statistical distribution, where the resulting curve is distorted and tends to be skewed to the left or right. The skewness value (*S*_k_) can be quantified by representing the difference between a given distribution and a normal distribution, as follows^50,61,62^:

1 $${S}_{k}=\frac{\frac{1}{n}\sum {({x}_{i}- \bar{x} )}^{3}}{{\sqrt{\frac{1}{n-1}\sum {({x}_{i}- \bar{x} )}^{2}}}^{3}}$$

where *n*, ${x}_{i}$, and $\bar{x}$ are the number of data points, independent data variables, and mean, respectively.

Kurtosis is a statistical measure that is usually used to assess the peak levels or flatness in a distribution. A high kurtosis value describes a distribution with special peaks and large tails, while a low kurtosis value represents a flatter distribution. The kurtosis value (*K*_u_) can be calculated by^50,63^:

2 $${K}_{u}=\frac{\frac{1}{n}\sum {({x}_{i}- \bar{x} )}^{4}}{{\left(\frac{1}{n}\sum {({x}_{i}- \bar{x} )}^{2}\right)}^{2}}-3$$

where 3 is the value for standard normal distribution.

In some cases, chi-square (*χ*^2^) can be used as a test for normality. To implement it, a normal distribution of the observed data becomes the null hypothesis for the expected value. *χ*^2^ can be described as^64^:

3 $${\chi }^{2}=\sum_{i=1}^{k}\frac{{\left({O}_{i}-{E}_{i}\right)}^{2}}{{E}_{i}}$$

where ${O}_{i}$ and ${E}_{i}$ are the observed and expected values, respectively. In the power-divergence (*P*_D_), the fit of the model can be assessed by comparing the expected frequencies for each outcome with the observed frequencies from our sample using^65^:

4 $${P}_{D}=\frac{2}{\lambda (\lambda +1)}\sum_{i=1}^{k}{O}_{i}\left[{\left(\frac{{O}_{i}}{{E}_{i}}\right)}^{\lambda }-1\right]$$

where $\lambda$ is a real-valued parameter that is chosen by the user. In this experiment, the employed power-divergence statistic is Pearson ${\chi }^{2}$ statistic with $\lambda \hspace{0.17em}=\hspace{0.17em}$1.

In statistics, the Jarque–Bera (*J*_B_) test is a normal distribution test of sampled data. This method investigates whether the sample data have any skewness and kurtosis compared to a normal distribution. Here, the statistical test of *J*_B_ is defined as^66–68^:

5 $${J}_{B}=\frac{n}{6}\left({S}_{k}^{2}+\frac{1}{4}{\left({K}_{u}-3\right)}^{2}\right).$$

For the normality test (*K*^2^), the quality of the fit is measured by a Gaussian function in a normal distribution based on D'Agostino-Pearson approach using^69^:

6 $${K}^{2}={Z}^{2}\left(\sqrt{{b}_{1}}\right)+{Z}^{2}\left({b}_{2}\right)$$

where $Z\left(\sqrt{{b}_{1}}\right)$ and $Z\left({b}_{2}\right)$ are the normal approximations to the sample estimates of skewness and kurtosis, respectively.

For the Gini index (*G*_I_), a statistical dispersion is measured in most cases. This method was also reported being able to perform an autofocus image scanning in holographic imaging^52,56,70,71^. The *G*_I_ can be calculated as^54–56^:

7 $${G}_{I}=1-2\sum_{k=1}^{N}\frac{{a}_{\left[k\right]}}{Sum\left(C\right)}\left(\frac{N-k+0.5}{N}\right)$$

where ${a}_{\left[k\right]}$ is the sorted data observation and *c* the data vector. In case *G*_I_ = 0, it indicates that the data have an equal amount of energy among them. Whereas, for *G*_I_ = 1, the input data have the highest inequality. In other words, the sparsest data are concentrated only in one element of energy.

All the statistical metrics that are briefly described above can be used to identify the “sharpest” image. The calculated results in Fig. 2b show that the wave propagation distribution analysis could produce the sharpest image in the *z*-propagated estimation from the angular spectrum algorithm by finding either global maxima (i.e., in the chi-square, power divergence, Jarque–Bera, kurtosis, normality test and Gini index) or global minima (i.e., in the skewness) of the metric values. Despite the gap between the USAF sample and the CMOS sensor, both global maxima and minima can be found as indicated by the yellowish dashed circle in Fig. 2b, in which the best in-focus position of the sample was found at *z* = 1.63 mm above the sensor. This outcome has therefore demonstrated that the optimum in-focus position of an object in the *z*-plane could be determined without any additional processing step prior to the angular spectrum or distribution analysis process.

**Figure 3:**
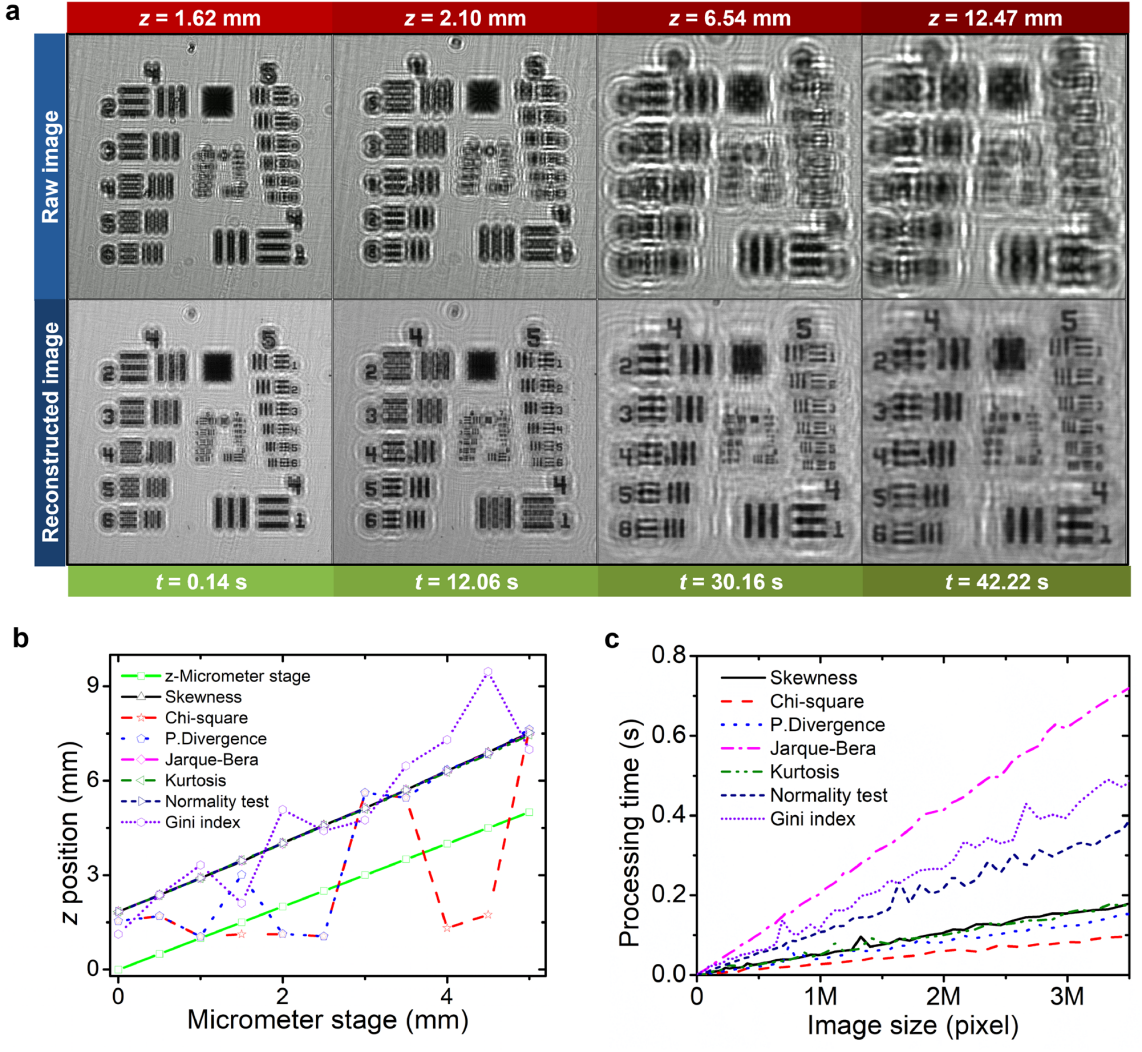
Demonstration of parfocal feature in a portable holographic microscope using a USAF target, which was moved along the *z*-axis. (**a**) Selected frames from a real-time video of continuous USAF sample movement in *z*-direction, showing a growth in diffraction and interference patterns along with the increasing sample-to-sensor distance. The parfocal feature for lensless microscopes was revealed from the reconstructed images, which have been processed using angular spectrum method and wave propagation distribution analysis. The computationally reconstructed USAF images could stay in-focus during the change of sample position in *z*-direction. The figure was generated using Python 2.7.14 (https://www.python.org) and Opencv 3.4.1 (https://opencv.org). (**b**) Comparison result between our statistical computation based on different distribution analysis methods and the manual step of the micrometer stage (green line). (**c**) The processing time needed to complete the computation for image reconstruction using various statistical distribution methods in respect to the incremental size of the region of interest on the observed image. (**b**) and (**c**) were created employing OriginPro 2019b (https://www.originlab.com).

To test the reliability of our autofocus approach as well as to manifest the computational parfocal feature in our lensfree microscope, the inspected USAF target was then moved continuously in *z*-direction using an *x–y–z* micrometer stage (Fig. 1b), in which several frames of the raw data and their reconstructed results from the recorded videos are displayed in Fig. 3a, respectively. For the raw images, the diffraction and interference patterns grew with increasing *z*-position as a result of the incrementally increasing distance between USAF sample and CMOS sensor. However, once the angular spectrum method and wave propagation distribution analysis have been applied to each frame from those sequentially captured images, the reconstructed USAF images were able to be kept in-focus, even when the sample was moving vertically in *z*-direction. This effect is similar to the feature of a parfocal lens in conventional microscopes. A time-lapse video demonstrating our nonmechanical parfocal function in the USAF target experiment is presented in Supplementary Materials (Video S1).

Our study has revealed that the histogram parameters were capable of distinguishing the difference between the clear and distorted images. By analyzing the density and distribution of pixel intensities, it was found that the crisp and sharp images mostly possessed non-symmetric distributions or were in “skewed” conditions. Since the basic principle of lensless in-line holographic microscopy is to capture the shadow image from the sample, the sharpness and crispness of the object can be analyzed from the shadow edge. In other words, how the intensity is distributed at the shadow edge may discriminate the object of interest from its background. From the *z*-micrometer stage movement experiment, skewness, Jarque–Bera, kurtosis, and normality test appeared to be promising to differentiate the sharpness and blurriness of the image in the holographic microscopy (Fig. 3b). Like the feature in a parfocal lens, these methods enabled the finding and maintaining of the sharpest image when the object changed its position without losing the focal point, resulting in the same gradient of *z*-position changes as the mechanical movement of the *z*-stage. Skewness was able to find the sharpest image independent on its origin (i.e., either from bright-field or dark-field microscopy). In that approach, the crispest image was found by searching for the skewed values that tended to be negative for bright field microscopy or positive for dark field microscopy. Meanwhile, the other methods (i.e., Jarque–Bera, kurtosis, and normality test) tended to find the largest distance from the normal distribution. On the other hand, weaknesses were found in the chi-square, power-divergence and Gini methods because they produced more than one local maximum at different *z*-positions, which resulted in ambiguities in determining the focus position (i.e., several peaks were produced along the focus position).

In general, a simple but powerful algorithm offers an enormous advantage in statistical computing. The use of distribution analysis in this system does not burden the central processing unit (CPU) usage. For all the conducted experiments in this report, a standard laptop with i5 processor of 2.2 GHz and random-access memory (RAM) of 8 GB was employed without graphics processing unit (GPU) acceleration, where the processing time required to calculate and compare the best in-focus value was moderately short (e.g., < 0.2 s for an image with size of 1 MP as indicated in Fig. 3c). Here, regardless of their accuracies, the chi-square and Jarque–Bera statistics have been observed as the quickest and lengthiest processing methods, respectively. In the real-time image processing, the frame rate depends on the size of the region of interest. The smaller area of interest, the shorter the computation time. Thus, the process requirements can be flexibly adjusted. For instance, in case of tracking a specific moving cell, the region of interest can be concentrated around that cell only, resulting in an even faster image processing speed. Meanwhile, if the monitoring should cover a wider field-of-view, the region of interest can be enlarged, hence the condition of that cell dynamic can be compared with those of other surrounding cells. During their cultures, the cells often grow as agglomerates at several different spots. Nonetheless, this rapid processing is achieved due to the fact that the image is directly calculated without requiring any pre-treatment or training process like in machine or deep learning. The image reconstruction can be performed in real-time, allowing the sample in the video to move along the *z*-axis, while simultaneously maintaining the sharpest image through a combined calculation of angular spectrum algorithm and wave propagation distribution analysis. However, the spatial resolution as well as the geometric magnification might change for different z-positions (see Fig. S5 in Supplementary Information). It should also be mentioned again that this analysis returns back the z-position of the tracked object.

Although sharp images could be obtained using the proposed system, few artefacts (i.e., twin images) are still apparent on the output images. These artefacts found in the images reconstructed with the angular spectrum method are attributed to spatial frequency aliasing, which is well known in the fast Fourier transform (FFT) algorithm^72,73^. Various methods (e.g., twin image reduction^38^ or deep neural network to perform cross-modality image transformation^74^) can be applied to holographic images for reducing speckle noises and image artefacts. Hence, images with a better signal-to-noise ratio can be produced. However, applying this technique will increase the required processing time since an additional iteration of post-processing step is needed causing a significant time-lag in the real-time monitoring process.

### 3D motion tracking of living cells

**Figure 4:**
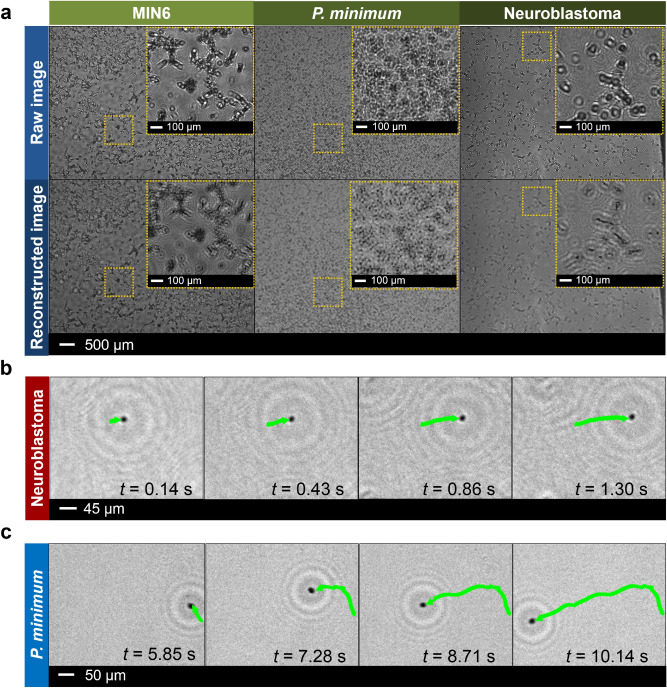
Real-time, label-free monitoring of biological cells using a parfocal autofocus-featured lensless microscope inside a sealed incubator. The cultured cells were placed in petri dishes. (**a**) Raw and reconstructed images of three different living cells (i.e., MIN6 cell line, *P. minimum*, and neuroblastoma (SH-SY5Y)). Motion tracking of (**b**) neuroblastoma and (**c**) *P. minimum* cells in *x*- and *y*-directions on a stable in-focus reconstruction mode. The green arrow indicates the moving direction of the inspected cells. The images were generated using Python 2.7.14 (https://www.python.org) and Opencv 3.4.1 (https://opencv.org).

Having a reliable automatic program based on the angular spectrum method and wave propagation distribution analysis for parfocal features, we then applied our lensless microscope to real-time and long-term observations of various cell cultures (i.e., MIN6, neuroblastoma (SH-SY5Y), and microalgae *Prorocentrum minimum* (*P. minimum*) cells) inside a sealed incubator chamber (Fig. S3 in Supplementary Information), in which the cell motility became the main interest of study (Fig. 4a–c). The experiments were performed from several days up to more than a week at a stable temperature resulting in high-quality images of the cells with the automated best in-focus position, even when they were actively moving. All the recorded videos containing the raw data and the reconstructed results of SH-SY5Y and *P. minimum* cells are presented in the Supplementary Materials (Videos S2 and S3, respectively).

From the obtained results, it was demonstrated that our lensless holographic microscope was able to observe and clearly differentiate the cultured cells of various shapes in a petri dish (Fig. 4a, first row). Initially, all cells appeared to have a similar circular or roundish form in the raw images, where the roundish occurrence was caused by diffraction. However, after applying the parfocal autofocus features to the reconstructed images, different irregular shapes of the MIN6 and neuroblastoma cells as well as the triangular oval-round form of the *P. minimum* cells could be clearly distinguished (Fig. 4a, second row). Fig. S4 in Supplementary Information depicts proliferation of MIN6 cells and formation of cell clusters during the long-term culture inside the incubator. These preformed clusters are the first step in the formation of spherically shaped pseudo-islets. Being able to monitor those cells in real time, we could then plan other experiments to gain more insights on cell growth mechanism. However, a deep bio-analysis was not considered as a focus in this study. This paper has been concentrated on the technical proof-of-concept for the development and integration of parfocal autofocus feature into portable LED-based lensless microscope equipped with CMOS image sensor.

**Figure 5:**
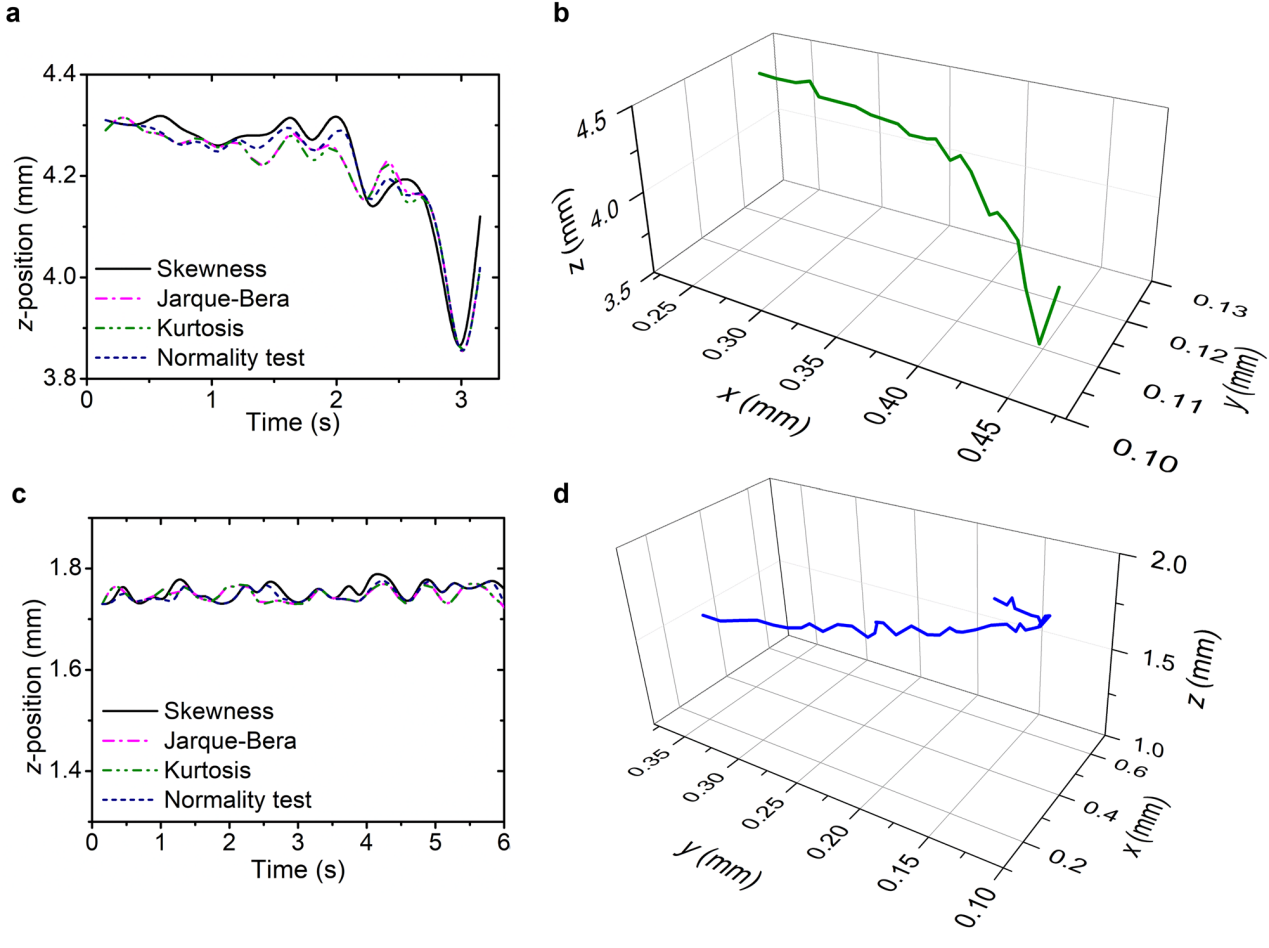
3D motion tracking of living neublastoma and *P. minimum* cells using a parforcal autofocus-featured lensless microscope. (**a**) In-focus *z*-position and (**b**) 3D trajectory of an actively moving neuroblastoma cell performing a diving motion. (**c**) In-focus *z*-position and (**d**) 3D trajectory of a tracked *P. minimum* cell having a drifting motion. Different wave propagation distribution analysis methods (i.e., skewness, Jarque–Bera, kurtosis, and normality test) were evaluated to find the best in-focus positions for both cells. All the figures were plotted using OriginPro 2019b (https://www.originlab.com).

It should be noted that these computational approaches can be activated directly from the beginning of the experiments to automate and provide a more accurate image reconstruction process in real-time, especially when dealing with a large amount of images or a long video duration. Furthermore, in comparison to the conventional optical microscopes that have a small field-of-view and depend highly on the field number and total magnification, our developed method allows the lensfree imaging system to produce very sharp images with a larger field-of-view (i.e., up to 5.7 mm × 4.3 mm depending on the employed image sensor). From the cell motion tracking experiments, this system was capable of delivering not only two-dimensional (2D) information of cell movement in *x*- and *y*-axes, but also the cell position in *z*-direction yielding a complete three-dimensional (3D) information dataset (Figs. 4b,c and 5a–d).

In Fig. 4b, the tracked neuroblastoma cell seemed to only swim forward along the *x*-axis, without actively changing the movement direction. To obtain 3D information of the cell trajectory, we implemented our method of cell tracking in the *z*-direction. According to the results from *z*-plane movement during USAF target experiments with a moving micrometer stage (Fig. 3b), we decided to implement only four different statistical methods (i.e., the skewness, Jarque–Bera, kurtosis, and normality test) for enabling the acquisition of the *z*-position of the cell. By combining the 2D movement tracking in *x–y*-direction and the *z*-value obtained from those calculation methods, our system could gain 3D trajectories of the neuroblastoma cell movements while keeping up with the ever-changing focus position (Fig. 5a). It was finally discovered that the cell was actually moving not only in *x*-axis but also in the *z*-direction within a range of up to 500 µm (Fig. 5b), resulting in observation of a diving motion. Nonetheless, this value is not the limit for our system. According to our previous USAF target experiments with a moving stage, an even larger *z*-trajectory range of up to 5 mm can be easily resolved.

In case of *P. minimum* cell tracking, although the cell was only moving within a range of 100 µm in *z*-direction (Fig. 5c), it was actively making abrupt changes in its motion direction along the *x–y*-axes, demonstrating a drifting motion at the beginning of the tracking (Figs. 4c and 5d). The cell activity was able to be further analyzed by monitoring its reconstructed 3D trajectory. Such a complete 3D position information with a sharp image is important for biologists and pharmacologists to investigate the reactions of the active target cells towards the introduced drugs during the development of novel pharmaceutical therapies for various specific diseases^75–77^. It was also demonstrated that the light emitted from a pinhole LED was harmless to the cells within a long-period observation^78^. The developed parfocal autofocus algorithm has rendered the lensless microscope very suitable as a cell culture monitoring device, especially in a harsh environment (e.g., inside the incubator with a specific temperature and humidity). A computational parfocal method combining the angular spectrum algorithm with the wave propagation distribution analysis has been proven to improve the qualities of the captured images from the USAF resolution test target and biological cells by comparing the image sharpness values of the objects of interest in different *z*-positions. Here, skewness and kurtosis methods appeared to be the most promising measures to perform discrimination between sharp and blurred images for finding the best in-focus plane in real-time holographic microscopy without any image pre-processing, which could then shorten the processing time significantly (Fig. 5a,c). Meanwhile, Jarque–Bera and normality test approaches provided good reconstruction results, since both are based on skewness and kurtosis methods.

In conclusion, the wave propagation distribution has been measured to analyze the intensity at all spatial frequencies from the distances of different objects to the CMOS sensor in the parfocal lensless holographic microscope having a pinhole LED-based light source. Here, a USAF resolution target and different biological cells have been investigated as the objects. The distribution data obtained by various statistical measures (i.e., skewness, Jarque–Bera, kurtosis, and normality) were then combined with the angular spectrum method to enable an automated finding of the focal point. Best in-focus positions of objects can be analyzed even during their movement in all directions (*x*-, *y*-, and *z*-axes). This method enables the compact microscope to perform a parfocal autofocus feature by real-time processing with neither using any lenses nor involving optical adjustment and a complex data training and pre-processing step. The in-focus position also provides the *z*-distance between the sensor and the object, thus also obtaining the 3D position and movement of the biological cells (i.e., MIN6, neuroblastoma, and *P. minimum* cells) inside the incubator, allowing long-term observations (more than a week). In the future, a deeper analysis of the influence of refractive properties of different media in the wave front propagation will have to be carried out to acquire a more accurate object-to-sensor distance, resulting in 3D live-cell motion tracking with even higher accuracy and error-reduced information.

## Methods

### Portable lensless microscope

The portable lensless holographic microscope consists mainly of an LED, a pinhole, a CMOS sensor, a 3D-printed housing, and electronics (Fig. 1a). Light produced from a GaN-based LED passes through a pinhole providing a partially coherent light source. It then impinges on the sample or object of interest, producing an interference pattern of the sample. The pattern is captured as a holographic image by a CMOS image sensor consisting of an arranged array of light-sensitive photodiodes (photocells). This component can detect brightness values and generate their corresponding bright or dark pixels. The denser is the photocells in an image sensor, the higher the spatial resolution of the produced image will be. This microscope is equipped with a monochrome CMOS sensor to detect light intensity without performing a color separation. The sample needs to be placed very close to the sensor and illuminated by light with an optimum coherence to obtain a highest possible spatial pixel resolution (Fig. 1b). Several extra features are also added in our microscope to improve the sample observation accessibility (i.e., an embedded computer, a Python-programmed data socket communication interface, and a cloud storage library). The embedded machine operates the light source, controls the camera of the CMOS sensor, and transmits the images using a wireless local area network connection to another computer or a smartphone for viewing and processing purposes. The Python data socket communication interface serves as a communication platform between the back-end system and the computer or smartphone. To perform a long-term monitoring, a cloud storage library is also inserted in our Python code. This library will send the images to the cloud data storage automatically, enabling observation, detection, and quantification of cells at anytime and anywhere.

Apart from the coherence optimization measurements that used different setups of LED and pinhole, all other experiments conducted in this work (for USAF and cell assessments) employed a blue LED with a wavelength of 467 nm as a light source. A 100 μm pinhole made of black oxide-finished stainless steel was adopted and placed over the image sensor to increase the spatial coherence of the LED (Fig. 1a,b). A CMOS camera with a field-of-view of 5.7 mm × 4.3 mm, a pixel array of 2592 × 1944, and a pixel size of 2.2 μm × 2.2 μm was integrated into the lensless microscope to capture the holographic images. A USAF 1951 resolution test target and various cells were used as samples to be investigated.

To find a compromise between spatial coherence and light intensity, the LED light source has been set to have a distance of 60 mm to the CMOS image sensor. This distance is estimated by calculating the first minimum from the complex degree of coherence produced by the pinhole LED, which corresponds to the resolution limitation through imperfect spatial coherence and inverse square law of the light intensity reduction by distance^79^. Placing the light source near to the sensor will reduce spatial coherence and therefore the resolution. Meanwhile, increasing the LED-to-detector distance will reduce the light intensity resulting in lower signal-to-noise ratio.

The employed blue LED with pinhole has an optical power density of 0.257 µW/cm^2^, which is much smaller than that without pinhole (i.e., 298 µW/cm^2^). Besides optical power, the exposure time was also investigated. For a real-time observation, a very short exposure time of the individual image frame has to be used within the setup. Therefore, it was set to be 0.1 s for each frame during continuous image acquisition. The gain value of the CMOS sensor adjusts the analog signal amplification. To obtain the optimum values of brightness and image capturing speed, the combination of exposure time and gain has been varied (see Figs. S6b and c in Supplementary Information). In our experiment, while we set the gain level at a value of 24, our lensless microscope was able to capture multiple frames per second with optimum brightness.

### Parfocal autofocus method

A fast, passive autofocus method is used to obtain a parfocal effect in the lensless microscopy. In contrast to active autofocus, which depends on the physical characteristics of the system (i.e., lens properties and distance among the optical parts), passive autofocus employs an image processing and a computational analysis based on the information of the captured sample image^49^. Here, a series of images captured at multiple *z*-positions is processed by calculating the feature value of the sharpness to establish the sharpest image and a correct focal position. The passive autofocus is more reliable for our lensless microscope, since it does not rely on the lens usage and any mechanical moving parts. Nevertheless, it depends highly on the optimum setup arrangement of the light source, image sensor, algorithm complexity, and computational performance.

### Automatic image reconstruction

The image captured by the CMOS sensor is defined by the area, occupied by the sample laid directly over it. A shadow image of the object is produced by the active pixel sensor or photodiode inside the camera chip. When a lens is attached onto the CMOS camera, the lens will gather and focus the light reflected from the object and subsequently direct it to the photodiode, enabling a sharp and crisp image creation. However, the focal length from the lens to the sample causes a sufficiently large gap between the CMOS, lens, and object, which decreases the field-of-view of the object. Thus, a lensless microscope that does not use any lens can minimize the gap between the CMOS and the object, providing a larger field-of-view. However, in the CMOS sensor, some transparent protection layers still exist above the active pixel sensor to filter, e.g., UV and infrared lights, to protect the photodiode, and to encapsulate the wire bonding. As consequence, the CMOS sensor cannot capture sharp images of the sample, especially for small scale objects, but rather produces a holographic image because the field changes due to the wave propagation. This phenomenon is related to the Rayleigh criterion of resolution and Abbe’s diffraction limit, where the created in-between distance affects the smallest individual object dimension that can be resolved. The image resolution depends on the wavelength of the light source, sample-to-sensor distance, and the diameter of the aperture.

The angular spectrum method is used to address this problem by reconstructing the wavefront to the desired *z*-position. Three key steps are needed to implement this algorithm. First, the field ${U}_{(x,y,{z}_{0})}$ is decomposed by transforming the image (*x*, *y*) in the plane ${z}_{0}$ into 2D plane waves via a spatial Fourier transform. Second, a complex notation of each plane wave is propagated into a 3D monochromatic plane wave plane in ${z}_{n}$ position. The wave vector of *z* depends on the sign, which is determined by whether the field propagates in positive *z* (above the sensor) or negative *z* direction (below the sensor). Third, the Fourier transform of the plane wave is inversed to the original domain to find the image reconstruction ${U}_{(x,y,{z}_{n})}$, as follows:

8 $${U}_{(x,y,{z}_{n})}={FT}^{-1}\left\{FT\left\{{U}_{(x,y,{z}_{0})}\right\}\left({f}_{x},{f}_{y}\right){e}^{2\pi i{z}_{n}\sqrt{{\frac{1}{\lambda }}^{2}-{f}_{x}^{2}-{f}_{y}^{2}}}\right\}(x,y)$$

where *f*_x_ and *f*_y_ are the spatial frequencies of 2D plane waves (*x*, *y*), respectively. Finally, the angular spectrum algorithm is completed by conducting all those three steps and adding all the propagated plane waves, allowing the calculation of the total propagated field using Eq. (8).

### First-order histogram analysis

The histogram of an image represents the distribution of the intensity, which is described by a statistical function related to the number of pixels in the whole image^62^. A histogram constructed from a grayscale image holds intensity values *I*(*u*, *v*) in a range of:

9 $$I\left(u,v\right)\in [0, k-1]$$

with intensity *I* for each pixel having a value of intensity range *k*. In the first-order histogram, histogram $H\left(i\right)$ represents the intensity levels of the image region and can be defined as:

10 $$H\left(i\right)=C\left\{\left(u,v\right)|I\left(u,v\right)=i\right\}$$

where *C* is the cardinality of a certain number set of pixels.

In holographic image processing, the sharpness of an image can be calculated by analyzing the data distribution. In our experiments, the intensity distribution is used to express the image sharpness level (i.e., level of blurriness or sharpness from the image), which can be found by analyzing its normality or non-normality level. Thus, the sharpest image along the wave propagation can be predicted. The closer the shape of image intensity distribution to the Gaussian curve is, the blurrier the image will be. On the other hand, the highest difference between the distribution shape and the Gaussian curve will yield the sharpest reconstructed image. Several types of data distribution analysis (i.e., skewness, chi-square, power divergence, Jarque–Bera, kurtosis, normality test, and Gini index) can be expressed as the distributional parameters to estimate and measure the inequality in the frequency distributions.

In general, a normal distribution will produce a classic and symmetrical bell-shaped curve. The frequency data distribution (${f}_{(x)}$) can be calculated by

11 $${f}_{(x)}= \frac{1}{\sigma \sqrt{2\pi }}{e}^{-\frac{1}{2}{(\frac{i- \bar{x} }{\sigma })}^{2}}$$

with

12 $$\sigma =\sqrt{\frac{\sum {({x}_{i}- \bar{x} )}^{2}}{n}}$$

where $\bar{x}$ and $\sigma$ are mean and standard deviation, respectively.

### Cell culture

Three different types of biological cells (i.e., MIN6, neuroblastoma (SH-SY5Y), and microalgae *Prorocentrum minimum* (*P. minimum*) cells) were cultured as objects of interest for the developed parfocal lensless holographic microscope. First, the insulin-secreting MIN6 cells (kindly provided by Jun-Ichi Miyazaki) were cultured in Dulbecco's modified Eagle medium (DMEM) with a high glucose concentration (4.5 g/l) of 25 mM, a final glutamine concentration of 6 mM, 10% fetal bovine serum (FBS), and penicillin/streptomycin in a humidified atmosphere of 95% air and 5% CO_2_ at 37 °C^80^. MIN6 pseudo-islets were generated by culturing MIN6 cells under the same conditions as described. To avoid cell attachment on the surface of the plastic material and to promote the formation of cell aggregates, dishes for suspension cell culture were used.

Second, the neuroblastoma cells were taken from the commercially available cell line SH-SY5Y (ATCC CRL-2266). This subclone of the original clone SK-N-SH cell line originates from a human neuroblastoma bone marrow biopsy and cultured in DMEM high glucose with 10% FBS^81^. It is easier and less expensive alternative to primary neuronal cultures since it is able to be differentiated into neuronal cell. Incubation was set in a humidified atmosphere at 37 °C and 5% CO_2_ to seed the undifferentiated cell inside 12-well cell culture plates. The viable cells, showing an irregular shape, are adherent to the surface of the plates, while non-viable cells are more roundish in shape and cannot adhere well to the surface. Third, the *P. minimum*, which is a eukaryotic single-cell algae belonging to the superclass of Dinoflagellate, was used in this study. *P. minimum* cells typically possess a triangular oval-round shape with length and width ranging from 17 to 25 µm and 14–23 µm, respectively^82^. Moreover, they are equipped with two flagella structures leading to their capability of actively moving through a medium. *P. minimum* culture was conducted at 20 °C in a petri dish containing artificial seawater medium under a day/night cycle of 12/12 h.

### Additional information

Information regarding the limit of resolution of the lensless microscope, the intensity simulation, the imaging setup for long-term cell culture observation, the imaged object size and distance relationship, and the light source properties can be found in the Supplementary Information. The recorded videos of moving USAF 1951 target, neuroblastoma, and *P. minimum* cells can be seen in Supplementary Materials.

## Supplementary Information

Supplementary Information.

Supplementary Video 1.

Supplementary Video 2.

Supplementary Video 3.

## Data Availability

The data that support the figures within this paper and other findings of this study are available from the corresponding authors upon reasonable request.

## Code availability

The code that supports the lensless microscopy monitoring and image analysis of this study is available from the corresponding authors upon reasonable request.
