# Supplementary material for: Nonmechanical parfocal and autofocus features based on wave propagation distribution in lensfree holographic microscopy
Source: Sci Rep. 2021 Feb 5;11:3213. doi: 10.1038/s41598-021-81098-7 (PMC7865004; doi:10.1038/s41598-021-81098-7)
Supplement: Supplementary file 1 — Supplementary Information. [file 41598_2021_81098_MOESM1_ESM.docx]

**Supplementary Information**

**Nonmechanical parfocal and autofocus features based on wave propagation distribution in lensfree holographic microscopy**

Agus Budi Dharmawan^1,2,3,*^, Shinta Mariana^1,2^, Gregor Scholz^1,2^, Philipp Hörmann^4^, Torben Schulze^5^, Kuwat Triyana^6^, Mayra Garcés-Schröder^1,2^, Ingo Rustenbeck^5^, Karsten Hiller^4^, Hutomo Suryo Wasisto^1,2,*^, Andreas Waag^1,2,*^

^1^ Institute of Semiconductor Technology (IHT), Technische Universität Braunschweig, Hans-Sommer-Straße 66, Braunschweig 38106, Germany

^2^ Laboratory for Emerging Nanometrology (LENA), Technische Universität Braunschweig, Langer Kamp 6, Braunschweig 38106, Germany

^3^ Faculty of Information Technology, Universitas Tarumanagara, Jl. Letjen S. Parman No. 1, Jakarta 11440, Indonesia

^4^ Institute for Biochemistry, Biotechnology and Bioinformatics, Braunschweig Integrated Centre of Systems Biology (BRICS), Technische Universität Braunschweig, Rebenring 56, Braunschweig 38106, Germany

^5^ Institute of Pharmacology, Toxicology and Clinical Pharmacy (IPT), Technische Universität Braunschweig, Mendelssohnstraße 1, Braunschweig 38106, Germany

^6^ Department of Physics, Faculty of Mathematics and Natural Sciences, Universitas Gadjah Mada, Sekip Utara PO Box BLS 21, Yogyakarta 55281, Indonesia

^*^ Corresponding authors.

E-mails: a.dharmawan@tu-braunschweig.de (A.B.D.); h.wasisto@tu-braunschweig.de (H.S.W.); a.waag@tu-braunschweig.de (A.W.); Phone: +49 531 391 3167

**Contents**

[**1. Limit of resolution for lensless microscope S2**](#_Toc58378212)

[**2. Intensity distribution simulation S3**](#_Toc58378213)

[**3. Imaging setup for long-term cell culture observation S4**](#_Toc58378214)

[**4. Imaged object size and distance relationship S6**](#_Toc58378215)

[**5. Light source properties S7**](#_Toc58378216)

# Limit of resolution for lensless microscope

From the conducted experiments using a USAF 1951 resolution test target, we could identify the limit of resolution for the developed lensless holographic microscope as depicted in **Fig. S1**. Here, four elements consisting of vertical and horizontal bars in the group 7 have become the main objects of interest. Thus, their intensity profiles were evaluated. The element 3 in the group 7 having a line width of 3.1 µm could be clearly resolved in the reconstructed image. Next, without any additional post image processing, the highest resolution of the microscope was obtained from the element 4 in the group 7, where both vertical and horizontal bars with a width of 2.76 µm could be resolved as sharp patterns. Meanwhile, for the element 5 in the group 7, although the 2.46 µm wide bars could still be recognized, they were not clear enough to be resolved. This can also be seen from their intensity profiles (shown in green). However, after performing an additional post image processing (i.e., finding the optimum threshold to distinguish both the high and low intensities), the structures were then able to be clearly resolved. The smallest element in the group 7 (i.e., element 6 with a bar width of 2.19 µm, shown in red) is difficult to be recognized. This has been indicated by their intensity pattern, which do not represent the real structures. Moreover, the size of this element 6 is also slightly smaller than that of one pixel area in the employed CMOS image sensor (i.e., 2.2 µm). Based on these measurement results, the best resolution for our system is 2.76 μm.


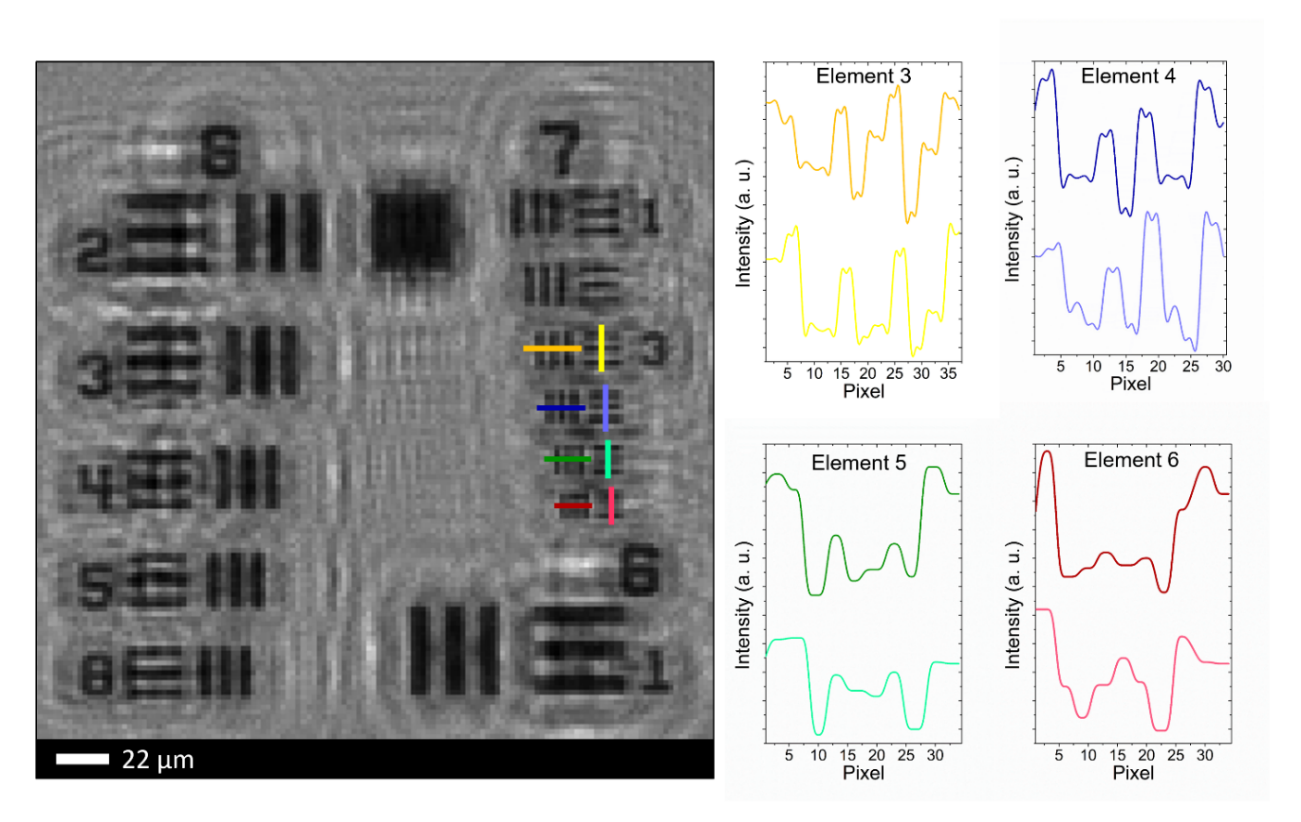


**Fig. S1** The raw image of a USAF resolution test target showing groups 6 and 7. The intensity profiles were evaluated for four elements (i.e., elements 3 - 6) in the group 7 with bars size ranging from 3.1 µm down to 2.19 µm, respectively. In this experiment, the target was placed directly above the CMOS image sensor. The USAF figure was created using Python 2.7.14 (<https://www.python.org>) and Opencv 3.4.1 (<https://opencv.org>), while the intensity profiles were plotted employing OriginPro 2019b (<https://www.originlab.com>).

# Intensity distribution simulation

The level of blurriness or sharpness of an image can be simulated by analyzing the level of normality or non-normality of the intensity distribution, as depicted in **Fig. S2**. The image sharpness was calculated based on the differences between the data distribution and the Gaussian curve. The simulation process was performed by employing a non-negative intensity value as an input. Hence, the simulation result is zero if all the data are identical and will increase as the data become more diverse. To evaluate their performances, seven statistical approaches were used in the simulations (i.e., Skewness, chi-square, P. divergence, Jarque-Bera, kurtosis, normality test, and Gini index). It should be noted that a special case was found in skewness method, in which two variations (i.e., positively and negatively skewed distributions) are present. Zero skewness value represented a normal distribution resulting in a classic and symmetrical bell-shaped curve. From the simulation results, the sharpest image is correlated with the lowest skewness-based distribution value. This value rises with the increasing Gaussian kernel size.


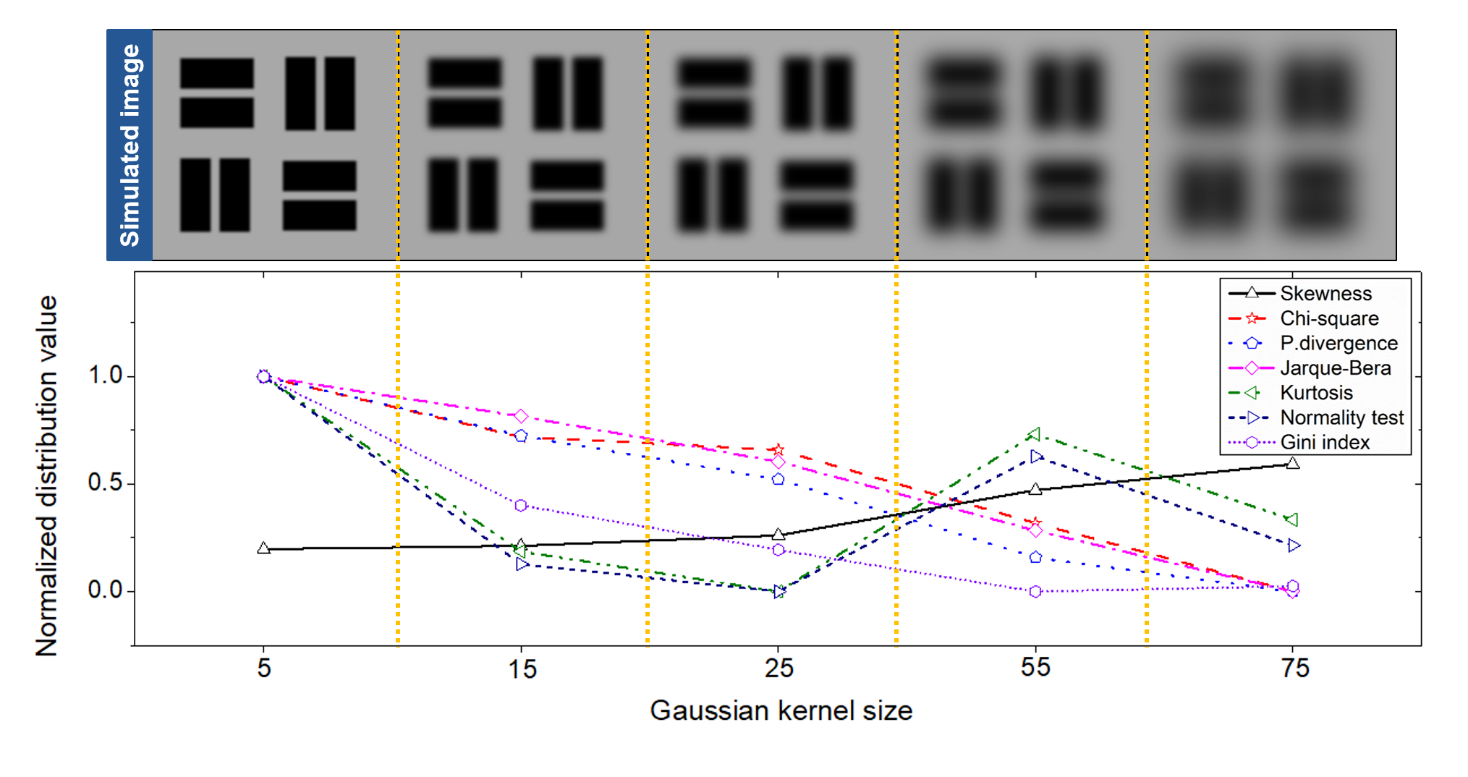


**Fig. S2** Simulated images and their intensity distribution values evaluated by seven different statistical approaches. The image simulations were performed by implementing various Gaussian kernel sizes to obtain several images with different blurriness levels. The simulated images were created using Python 2.7.14 (<https://www.python.org>) and Opencv 3.4.1 (<https://opencv.org>), while the normalized distribution figure was made using OriginPro 2019b (<https://www.originlab.com>).

# Imaging setup for long-term cell culture observation

Cultures of three different biological cell types were investigated using the developed parfocal lensless microscope (i.e., MIN6, neuroblastoma (SH-SY5Y), and *P. minimum* cells). For all the conducted experiments, the cells remained inside a completely sealed incubator chamber during the measurements without any interruption. The monitoring process was controlled by an in-house software, where all the measured data were sent to cloud storage automatically without the need to open the incubator during the observation period (**Fig. S3**). To minimize the heating effect from the image sensor that might damage the cells as well as to control the temperature of the sensing area, the microscope was equipped with a module comprising a temperature sensor, a Peltier element, and a programmable power control. This embedded machine will read the temperature from the temperature sensor and simultaneously control the generated power, resulting in an active cooling. Hence, the temperature of the CMOS sensor could be kept similar to that of the incubator. The supplied power to the CMOS sensor was also carefully adjusted to avoid excessive heat and energy.


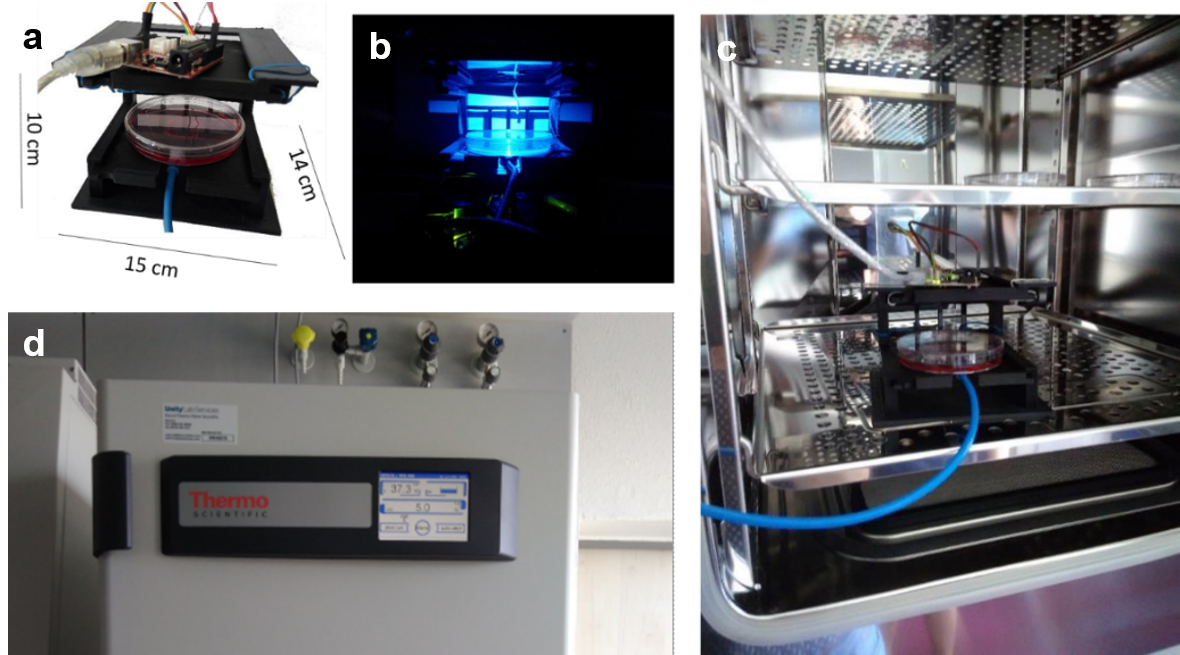


**Fig. S3** **a** The developed light-emitting diode (LED)-based lensless microscope with a parfocal feature during **b** active operation for observing cells in **c** a petri dish. The live-cell culture monitoring experiment was performed inside **d** a sealed incubator without any interruption.

To validate the imaging system for a long-term operation, an uninterrupted measurement was performed within seven days using the MIN6 cells inside a petri dish as an object of interest. The image was captured automatically every 15 min and stored to both local and cloud storages. In total, 672 images were produced during this experiment. Our autofocus feature enabled the development of clear and sharp images by performing a real-time automatic reconstruction for each image (**Fig. S4**). It could provide the sharpest images with a higher accuracy, avoiding possible errors that might occur when humans were involved in a manual processing for a large amount of images.


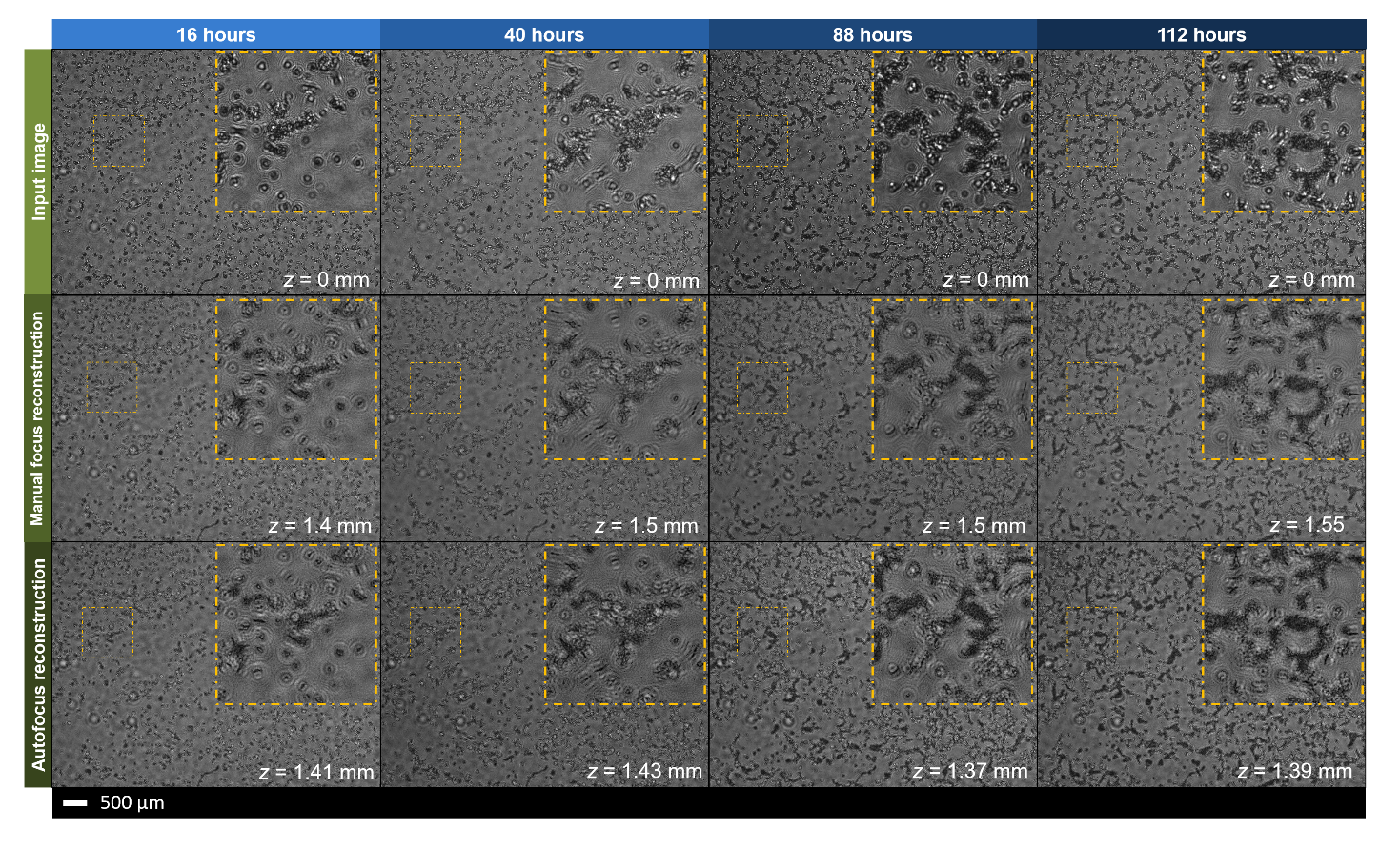


**Fig. S4** Long-term observation results of MIN6 cells inside a sealed incubator. The autofocus feature was able to reconstruct the sharpest images automatically for a large amount of data in real-time. The figures were generated using Python 2.7.14 (<https://www.python.org>) and Opencv 3.4.1 (<https://opencv.org>).

The videos of neuroblastoma and *P. minimum* cells were recorded at 7 fps with a maximum resolution of 2592 × 1944 pixels. The video sequence was reconstructed in real-time using the proposed autofocus and parfocal features. The cells were cultured inside a sealed incubator without any interruption. The videos of moving neuroblastoma (SH-SY5Y) and *P. minimum* cells within regions of interest are presented in Supplementary Materials as **Videos S2 and S3**, respectively.

# Imaged object size and distance relationship

**Fig. S5** shows a relationship between the object distance from the CMOS sensor and the reconstructed image magnification from the 556.8 µm object of USAF 1951 resolution pattern. The USAF target was moved along z-direction using an *x-y-z* stage, in which a CMOS sensor simultaneously captured the shadow images of the sample. The *z*-position was calculated using our parfocal method, while the size of the object was determined by measuring the width of dark area from a square bar in group 2. The result shows that the size of the imaged object size has become larger with the increasing sensor-to-sample distance. However, the increment of sample size in lensless not linear with the geometric magnification due to the geometric unsharpness phenomenon.


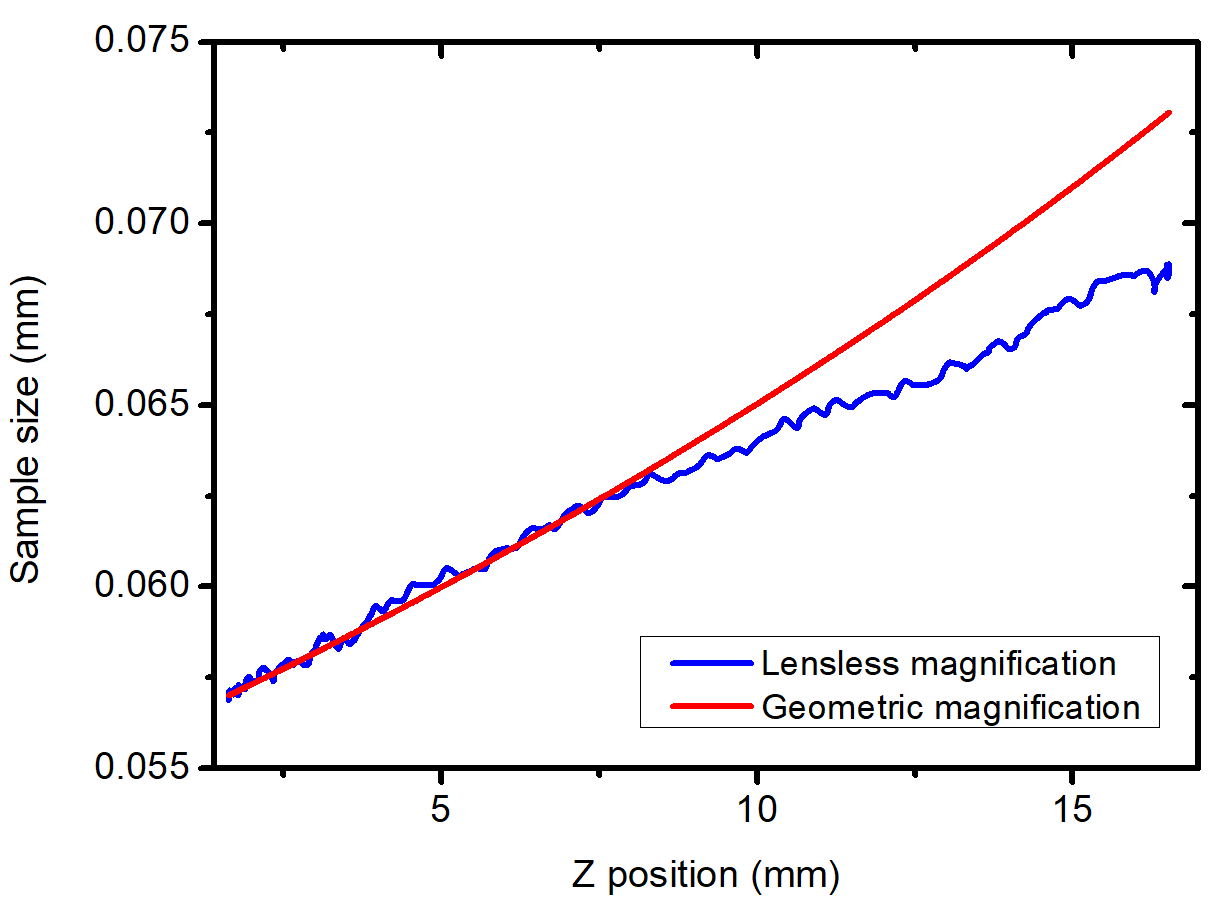


**Fig. S5** The size increment versus object-to-CMOS sensor distance. The result indicates that the magnification increases when the sample-to-sensor distance becomes larger. The figure was created using OriginPro 2019b (<https://www.originlab.com>).

# Light source properties

We compared the spatial coherence values of the employed blue LED having a size of 150 µm × 200 µm before and after being filtered with 100 µm pinhole. Based on the results shown in **Fig. S6a**, the temporal coherence remained the same for both light sources with different sizes of emitting areas. Different exposure times were investigated to find the optimum value of the gain level for the CMOS sensor (**Fig. S6b**). This optimization was necessary to obtain sufficient brightness and high speed of image capturing. Longer exposure time increases the brightness but at the same time also reduces the frame rate. Here, the gain function of the CMOS sensor can be used to amplify the readout signal from the pixel before it is stored as a digital pixel value. This will allow the system to acquire the appropriate brightness with a fast exposure time. In our experiment, we defined the gain value to be 24, which was the maximum value for the CMOS sensor. Moreover, the exposure time of 0.1 s was set to result in a fast image capturing process. **Fig S6c** depicts the images of USAF chart at three different brightness levels (i.e., 0.1, 0.65, and 0.98). The best image was obtained when the brightness value of 0.65 was used in our lensless microscope.


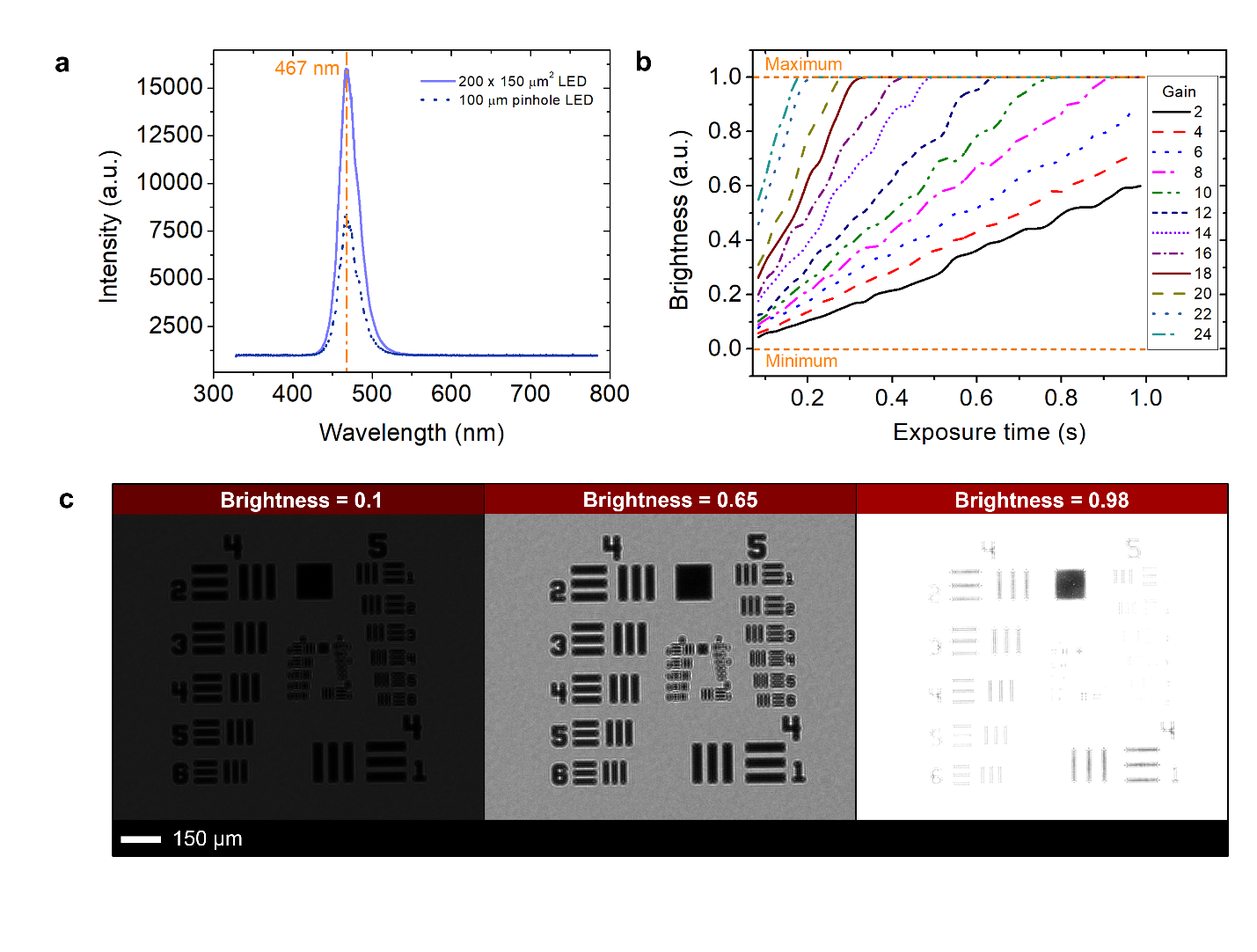


**Fig. S6** **a** Temporal coherence comparison between blue LED with and without pinhole. **b** Correlation between exposure time and brightness resulting in various gain levels. Figures **a** and **b** were created using OriginPro 2019b (<https://www.originlab.com>). **c** Captured USAF chart images at different brightness values. This figure was generated employing Python 2.7.14 (<https://www.python.org>).
